# Sequencing of two transgenic early-flowering poplar lines confirmed vector-free single-locus T-DNA integration

**DOI:** 10.1007/s11248-020-00203-0

**Published:** 2020-04-30

**Authors:** Birgit Kersten, Ana Paula Leite Montalvão, Hans Hoenicka, Cristina Vettori, Donatella Paffetti, Matthias Fladung

**Affiliations:** 1 Thünen Institute of Forest Genetics, 22927 Grosshansdorf, Germany; 2 Institute of Bioscience and Bioresources (IBBR), National Research Council (CNR), Via Madonna del Piano 10, 50019 Sesto Fiorentino, FI Italy; 3 Department of Agriculture, Food, Environment and Forestry, Agricultural Genetics Section, University of Florence, P. le delle Cascine 18, 50144 Florence, Italy

**Keywords:** *Populus*, Poplar breeding, Transgene-free, Early-flowering, Biosafety research

## Abstract

Next-generation sequencing (NGS) approaches are attractive alternatives to the PCR-based characterisation of genetically modified plants for safety assessment and labelling since NGS is highly sensitive to the detection of T-DNA inserts as well as vector backbone sequences in transgenic plants. In this study, two independent transgenic male *Populus tremula* lines, T193-2 and T195-1, both carrying the *FLOWERING LOCUS T* gene from *Arabidopsis thaliana* under control of a heat-inducible promoter (pHSP::*AtFT*) and the non-transgenic control clone W52, were further characterised by NGS and third-generation sequencing. The results support previous findings that the T-DNA was hemizygously inserted in one genomic locus of each line. However, the T-DNA insertions consist of conglomerations of one or two T-DNA copies together with a small T-DNA fragment without *AtFT* parts. Based on NGS data, no additional T-DNA splinters or vector backbone sequences could be identified in the genome of the two transgenic lines. Seedlings derived from crosses between the pHSP::*AtFT* transgenic male parents and female wild type plants are therefore expected to be T-DNA splinter or vector backbone free. Thus, PCR analyses amplifying a partial T-DNA fragment with *AtFT*-specific primers are sufficient to determine whether the seedlings are transgenic or not. An analysis of 72 second generation-seedlings clearly showed that about 50% of them still reveal the presence of the T-DNA, confirming data already published. To prove if unanticipated genomic changes were induced by T-DNA integration, extended future studies using long-range sequencing technologies are required once a suitable chromosome-level *P. tremula* reference genome sequence is available.

**Electronic supplementary material:**

The online version of this article (10.1007/s11248-020-00203-0) contains supplementary material, which is available to authorized users.

## Introduction

The European Union has established a number of legal frameworks regulating the practical use of genetically modified (GM) organisms, *i.e.* Directive 2001/18/EC on the deliberate release of GM organisms into the environment (European_Parliament_Council 2001). This strict legislation officially aims to ensure that the development of modern biotechnology, specifically including GM organisms, takes place in safe conditions (Davison 2010). However, these EU regulations unfortunately hamper, or even impede, the market introduction of GM plants, including trees (Custers et al. 2016; Fladung et al. 2012).

A GM organism is defined as an organism whose genetic material has been altered in a way that does not occur naturally by mating and/or natural recombination (Article 2 of Directive 2001/18/EC), but rather by means of genetic engineering. In addition, an organism is classified as GM when it is produced by the technique of genetic modification, even when the foreign DNA or RNA is no longer present in the genome (New_Techniques_Working_Group 2011; New_Techniques_Working_Group and Poudelet 2014). This regulation is important when a hemizygous GM plant is sexually propagated, i.e. either selfed or crossed with a non-GM plant. In both cases, offspring is produced with theoretically 75% (self-fertilization) or 50% (cross with non-GM) GM plants and 25% (self-fertilization) or 50% (cross with non-GM) plants without recombinant DNA, according to Mendelian segregation. Even if the group of *F*_1_-non-GM plants is undoubtedly free of any T-DNA inserts, this group will be considered as GM according to Directive 2001/18/EC.

In *Agrobacterium*-based transformation, T-DNA is inserted into the plant cell and integrated randomly somewhere in the genome (Fladung 1999; Forsbach et al. 2003; Kumar and Fladung 2001), although some exceptions have been reported (Brunaud et al. 2002; Zhang et al. 2007). The presence of T-DNA in the plant genome is routinely checked using “classical” molecular techniques such as PCR and Southern blotting (copy number), and also using TAIL-PCR and other techniques to unravel T-DNA flanking genomic sequences and locate the genomic position in the case of the availability of aligned genomes. For a comprehensive risk evaluation following the guidelines published by EFSA (EFSA_Panel_on_Genetically_Modified_Organisms 2011), additional molecular characterisation is needed, including putatively endogenous host gene interruption by the T-DNA insert and evaluation of the differences between inserted and junction sequences in genes known to encode toxins or allergens (Pauwels et al. 2015; Schouten et al. 2017; Yang et al. 2013). Additionally, the integration of partial T-DNA inserts and vector backbone sequences (outside of the main T-DNA insert), or even the occurrence of genomic rearrangements or mutations, has been reported in transgenic plants (Fladung 1999; Jupe et al. 2019; Pawlowski and Somers 1998; Wilson et al. 2006).

Next-generation sequencing (NGS) has been shown to be a highly sensitive approach for detecting T-DNA inserts as well as vector backbone sequences in transgenic plants (Guo et al. 2016; Holst-Jensen et al. 2016; Jupe et al. 2019; Park et al. 2015; Pauwels et al. 2015; Schouten et al. 2017; Yang et al. 2013). The NGS-based approaches are attractive alternatives to PCR-based characterisation methods for the safety assessment and labelling of GM plants (Guo et al. 2016) and for the detection of GM ingredients in processed products (Holst-Jensen et al. 2016; Li et al. 2017). The comprehensive molecular investigation using NGS provides an opportunity to identify and characterise additional unintended insertions and unknown GM events compared with the results from Southern blot analyses (Yang et al. 2013).

In earlier papers, we reported the genetic characterisation of the offspring following the crossing of two early-flowering independent transgenic poplar lines carrying the heat-inducible *FLOWERING LOCUS T* gene from *Arabidopsis thaliana* (pHSP::*AtFT*) with non-transgenic clones (Hoenicka et al. 2012, 2014, 2016). In PCR and Southern blot analyses, we clearly showed that about half of the *F*_1_ individuals still reveal the presence of T-DNA, while the second half does not (Hoenicka et al. 2014). Here, we sequenced and analysed the complete genome of the two transgenic parent lines by next and third-generation sequencing technologies (Ion Torrent, Illumina, nanopore) to identify the genomic T-DNA integration sites and to screen the sequences of the transgenic parent lines for potential T-DNA splinter and/or vector backbone sequences that can also be transmitted to the T-DNA-free fraction. Whereas long read sequencing by third-generation technologies such as nanopore are very useful to identify T-DNA integration sites and unravel the genomic structure of T-DNA insertions (Jupe et al. 2019), short reads are—due to low sequencing error rates—particularly well suited for the identification of potential short T-DNA or vector backbone splinters in transgenic lines (Li et al. 2017; Schouten et al. 2017).

Using bioinformatic analyses, we confirmed vector-free single-locus T-DNA integration in the two transgenic lines as previously determined by Southern blot analyses (Hoenicka et al. 2012, 2014, 2016). We were unable to detect any T-DNA splinters (Schouten et al. 2017) or vector backbone sequences in the genomes of the two transgenic parent lines. From these results, we can also conclude that the T-DNA-free *F*_1_ offspring of both crosses don’t reveal any T-DNA splinters and the aberrant phenotypes sometimes observed result from interspecific crossing rather than transgenesis (Hoenicka et al. 2014).

## Materials and methods

### Sanger sequencing of the T-DNA insert of pK2GW7_HSP_FT

The T-DNA vector pK2GW7_HSP_FT (Huang et al. 2005) and a related draft nucleotide sequence (the vector backbone sequence according to the sequence of the binary Gateway destination vector pK2GW7; Genbank accession JC487359) were kindly provided by O. Nilsson (Swedish University of Agricultural Sciences, Umeå, Sweden). Based on the draft sequence, four primer pairs were designed (Suppl. file 1) to amplify four overlapping vector fragments covering the T-DNA region (239 bp to 3144 bp from the 5-prime end of the left border to the 3-prime end of the right border).

PCR reactions were performed in 1 × reaction buffer BD (provided together with Taq-polymerase by DNA Cloning Service, Hamburg, Germany), 1.8 mM MgCl_2_, 200 µM dNTP-Mix, 0.4 µM of each primer, 0.125 µl Taq-DNA polymerase (5 units/µl) and 100 ng DNA (in a total volume of 25 µl). The PCR program was started with an initial denaturation for 2 min at 94 °C. Thirty-eight PCR cycles followed with 30 s at 94 °C, 45 s at 58 °C and 90 s at 72 °C. The reaction was completed by a final elongation step for 5 min at 72 °C. PCR products in Orange G-based loading buffer were made visible on 1.2% agarose gel in 0.5 × TBE buffer (100 V) stained with the DNA fluorescence additive Roti-GelStain (Carl Roth, Karlsruhe, Germany). For Sanger sequencing (StarSeq, Mainz, Germany), 1 µl PCR product was mixed with 1 µl sequencing primer and 5 µl H_2_O.

Based on the Sanger sequences, the draft vector sequence was mainly edited in the T-DNA region to create the nucleotide sequence of the vector pK2GW7_HSP_FT (9470 bp; GenBank accession MN379653).

### Plant material, culture and genetic transformation

The two early-flowering poplar lines, T193-2 and T195-1, were obtained through genetic transformation of the male (♂) poplar (*P. tremula* L.) clone W52 with pHSP::*AtFT FLOWERING LOCUS T* (*FT* from *A. thaliana* under the control of a heat shock promoter), as described previously (Hoenicka et al. 2012). The genetic transformation was carried out using the *Agrobacterium*-mediated approach (Fladung et al. 1997) with *Agrobacterium tumefaciens*, strain EHA105. For regeneration of transgenic plants, Woody Plant Medium (WPM) was supplemented with 0.01% Pluronic F-68 (Sigma P-7061, Steinhein, Germany), thidiazuron (0.01 μM) and antibiotics cefotaxime (500 mg l^−1^) for agrobacteria elimination, and kanamycin (50 mg l^−1^) for the selection of transgenic shoots.

Plants from the two clones were grown under aseptic conditions on solid McCown WPM (Duchefa M0220, The Netherlands) (Lloyd and McCown 1980) containing 2% sucrose and 0.6% agar (Agar Agar, Serva, 11396, Germany). Soil-potted plants were transferred to growth chambers (Weiss Technik, Reiskirchen, Germany) and cultivated under the following culture conditions: light period, 16/8 h (day/night); light intensity, 300 µE m^−2^ s^−1^ (lamps, Phillips TLM 140 W/33RS, Amsterdam, The Netherlands); relative humidity, 70% and temperature, 22/19 °C. After a culture period of 6–18 months in growth chambers, the transgenic plants were transferred to a standard S1 greenhouse and cultivated under natural daylight conditions.

### Induction of fertile flowers in pHSP::AtFT transgenic poplar plants

Fertile flowers were induced in a 6-month-old pHSP::*AtFT* seedling (*F*_1_ generation, ♂) (Hoenicka et al. 2014) according to a previously described protocol (Hoenicka et al. 2016). In short, plants were subjected to two culture phases in a growth chamber. During Phase 1 (P1; flower induction), heat treatments (40 °C, 90 min, 3–5 weeks, day/night: 22/16 °C, 16/8 h) were applied daily until initiation of flower development. During Phase 2 (P2; fertility induction), the poplar plants were cultivated for 8–12 weeks under cold conditions (day/night: 10/6 °C, 16/8 h).

### Crossing of a wildtype poplar with a pHSP::AtFT *F*_1_ seedling and molecular analysis

Crossings were carried out between a heat-induced early-flowering male pHSP::*AtFT* seedling (*F*_1_ generation derived from transgenic line T193-2 and wild type poplar (*P. tremula* L., clone W7) and a wild type female hybrid poplar (*P. tremula* L. × *P. tremuloides* Michx., clone Esch9). Twigs of the Esch9 clone were harvested in late winter and transferred to large glass vessels with running water located in the greenhouse under natural light and temperature regimes. Catkins with fertile female flowers developed in the following weeks and were fertilised with pollen harvested from a heat-induced early-flowering pHSP::*AtFT* seedling (*F*_1_). Seeds obtained (second generation) were cleaned of wool, germinated in a growth chamber and later transferred to the greenhouse.

Genomic DNA was extracted from the leaves of seedlings obtained from controlled crosses according to established protocols (Hoenicka et al. 2012, 2014). PCR analyses were carried out with *AtFT*-specific primers (For 5′-GTT GGA GAC GTT CTT GAT CCG-3′, Rev 5′-TCT TCT TCC TCC GCA GCC ACT-3′) with an annealing temperature of 62 °C, following a previously described procedure (Hoenicka et al. 2012).

### Ion Torrent sequencing of W52, T193-2 and T195-1

Genomic DNA was extracted from leaves of W52 (non-transgenic control), T193-2 and T195-1 and DNA extraction followed a standard protocol adapted from (Doyle and Doyle 1987) using 0.5–1.0 g leaf material and a modified extraction buffer (2% alkyltrimethylammonium bromide (ATMAB), 0.1 M Tris-HCl, 0.02 M disodium-EDTA (pH 8.0), 1.4 M NaCl, 1% PVP). The Ion Torrent sequencing platform was used for the shotgun sequencing of the total genomic DNA of W52, T193-2 and T195-1 samples using the Personal Genome Machine (PGM) Sequencer (Life Technologies, USA). Total genomic DNA (100 ng) was sheared using the Ion Shear Plus Reagents and used for preparing the sequencing library according to the Ion Xpress Plus gDNA Fragment Library kit (cat n. 4471252) following Ion Torrent PGM protocol (Life Technologies, USA). The resulting individual DNA libraries were quality checked and quantified using the Qubit 2.0 Fluorometer and the Qubit dsDNA HS Assay Kit following the manufacturer’s specifications (Life Technologies, USA). Following template preparation (amplification and enrichment) and Ion 318 Chip Kit V2 BC (cat n. 4488150, Life Technologies, USA) loading on the Ion Chef System (Life Technologies, USA) using the Ion PGM Hi-Q View Chef 400 kit (cat n. A30798), the Ion 318 Chip v2 was loaded on the PGM (Life Technologies, USA) and sequenced using the Ion PGM Hi-Q View Chef 400 (cat n. A30798, Life Technologies, USA) according to the manufacturer’s protocol.

### Illumina sequencing of T195-1

Genomic DNA was extracted from leaves of T195-1 as described above. A genomic library was generated and analysed by Illumina HiSeq 4000 sequencing (2 × 150 bp; Novogene, Beijing, China).

### High molecular weight DNA extraction and MinION sequencing of T193-2 and T195-1

Leaf samples (~ 90 mg) were collected into 2 ml Eppendorf tubes and frozen in liquid nitrogen. Samples were ground using the Bead Ruptor Elite (Bead Mill Homogenizer, OMINI International) with two stainless steel beads (5 mm) per tube at a speed of 2.10 m/s for 20 s. For the DNA extraction, we combined a pre-lysis sorbitol wash with a CTAB extraction [adapted from (Inglis et al. 2018)] and a final bead clean-up step.

The sorbitol wash buffer (100 mM Tris-HCl, 0.35 M Sorbitol, 5 mM EDTA pH 8.0, 1% (w/v) polyvinylpyrrolidone (molecular weight 40,000; PVP-40)) was freshly prepared and just before performing the extraction, 1% (v/v) β-mercaptoethanol was added. Sorbitol buffer (1 ml) was added to each sample. Samples were then mixed by inverting the tube five times and centrifuged at 3000 × g for 5 min at room temperature. The supernatant was carefully discarded. For sample lysis and DNA extraction, pre-heated (65 °C) CTAB buffer (100 mM Tris-HCl, 3 M NaCl, 3% CTAB (cetyl trimethylammonium bromide), 20 mM EDTA and 3% (w/v) polyvinylpyrrolidone (PVP-40; molecular weight 40,000), 2% sodium metabisulfite and 1% (v/v) β-mercaptoethanol (added just before use)) was added to the samples (600 µl per tube), mixed well and then incubated at 56 °C for 1 h. After cooling down at room temperature for 5 min, phase separation was performed twice by adding an equal volume of chloroform:isoamylalcohol (24:1) and centrifuging at 3000 g for 10 min at room temperature, after which the upper aqueous phase was carefully transferred to a fresh tube. Precipitation was performed by adding ice-cold isopropanol at 0.66 of the sample volume and samples were mixed by inversion and stored at − 20 °C for 1 h. DNA was pelleted by centrifugation at 13000 × g for 10 min at room temperature. Subsequently, the supernatant was removed and the pellets were washed in 1 ml 70% ethanol. Finally, the pellets were resuspended in 100 µl Tris-HCl containing 0.1 mg/ml DNase-free RNase A and incubated at 37 °C for 20 min.

Sera-Mag SpeedBeads were used to perform size selection (removal of small fragments) and purification of the samples (Schalamun et al. 2019). The previously extracted DNA samples were pooled together in a 1.5 ml LoBind Eppendorf tube and 0.8 V of a homogenised beads solution (10 mM Tris-HCl, 1 mM EDTA pH 8.0, 1.6 M NaCl, 11% PEG 8000, 0.4% beads (v/v)) was added to the tube and mixed by gentle flicking. The tube was mixed (HulaMixer) for 10 min, then briefly centrifuged and placed on a magnet. Once the solution was clear and the beads were on the back of the tube, the supernatant was discarded and the beads were washed twice with 1 ml of freshly prepared 70% ethanol. After the last ethanol removal, the tube was taken off the magnet and briefly centrifuged. After placing it back on the magnet, the last drops of ethanol were pipetted off. The beads were air dried for 20 s, after which the tube was removed from the magnet and 50 µl of pre-heated (50 °C) 10 mM Tris was added for elution. The tube was flicked to properly resuspend the beads and the tube was incubated for 10 min at room temperature. Finally, the tube was placed back on the magnet and, once the solution was clear, it was transferred to a fresh tube.

DNA purity and concentration were measured by a spectrophotometer (Nanodrop, 1000; Peqlab) and a fluorimeter (Qubit 3.0, dsDNA Broad Range Assay Kit; Thermo Fisher Scientific), respectively.

Library preparation as well as priming and loading the flow cell (version R9.4.1) were performed following the Ligation Sequencing Kit (SQK–LSK109) protocol from Oxford Nanopore Technologies (ONT) and sequencing was performed in the MinION device.

### Ion Torrent data analysis

All steps of the Ion Torrent data analysis were performed using the CLC Genomics Workbench (CLC GWB) v11.0 (QIAGEN, Germany) if not otherwise stated. The Ion Torrent reads of all three genotypes (W52, T193-2 and T195-1) were trimmed using the “trim reads” tool. The following parameters were set to “yes”: ambiguous trim (limit = 2); trim adapter list; discard short reads (minimum number of nucleotides in reads = 80). During the trimming step, the Ion adapter P1 was removed from the 3-prime end of the reads and Ion forward adapter sequences were removed from the 5-prime ends, if included (adapter sequences and information on the trimmed reads in Suppl. file 2).

To check for T-DNA integration into the genomes of the transgenic lines, the trimmed reads were stringently mapped to the complete sequence of the T-DNA vector pK2GW7_HSP_FT (MN379653) using the “map reads to reference” tool of the CLC GWB (default parameters but with a length fraction of 0.9, a similarity fraction of 0.95 and the output mode “create reads tracks”). Coverage plots were created from the read tracks using the “create mapping graph tracks” tool.

Using Ion Torrent data, the T-DNA insert was localised based on chimeric reads, which are reads that contain both a vector fragment and a genomic *P. tremula* fragment. To identify the chimeric reads, trimmed Ion Torrent reads of the transgenic lines were mapped to the vector sequence with the default parameters but with a length fraction of 0.3 and a similarity fraction of 0.98. Using the CLC GWB, all mapped reads were extracted and analysed by BLASTN with default parameters (but an e-value of E-5) to (1) genomic scaffolds of *P. tremula* v1.1 that were downloaded from Popgenie v3 (Lin et al. 2018; PopGenIE 2019; Sundell et al. 2015) and (2) the *P. trichocarpa* genome assembly v3.0 downloaded from Phytozome v12 (JGI Phytozome 2019; Tuskan et al. 2006). All reads with the highest BLAST hit identities above 93% were selected as chimeric reads.

### Illumina data analysis

The bioinformatic analysis of the Illumina data of T195-1 was performed using the CLC GWB v12.0 (QIAGEN, Germany) if not otherwise stated. Raw reads were trimmed using the “trim reads” tool (adapter, quality, ambiguity, terminal nucleotides and length trimming) with the following parameters: quality limit = 0.03; ambiguous limit = 2; automatic read-through adapter trimming = no; number of 5-prime/3-prime terminal nucleotides = 1; minimum read length = 80. Broken pairs were saved. For adapter trimming the following partial adapter sequences were used to identify 3-prime ends of reads for trimming: AGATCGGAAGAGCGTCGTGTAGGGAAAGAGTGT (universal_i5_rev_comp) and AGATCGGAAGAGCACACGTCTGAACTCCAGTCAC (index_i7_without_index_region).

To create a coverage plot, trimmed paired and single reads (orphans) were mapped to pK2GW7_HSP_FT (MN379653) as described above in “Ion Torrent data analysis”.

### Genome-wide screen for T-DNA vector splinters in T193-2 and T195-1 using short reads

A genome-wide screen for potential T-DNA vector splinters of at least 20 bp was performed for T193-2 and T195-1 using vector k-mers. For this purpose, the nucleotide sequence of pK2GW7_HSP_FT (Genbank accession MN379653) was in silico digested into k-mers of 20 bp length (shift of 1 bp) by applying the following UNIX command to the original vector sequence and 19 modified versions of the sequence (modification by 5-prime removing and 3-prime adding of 1 to 19 nucleotides): “grep -v ‘^ > ’ vector.fa| tr -d ‘\n’| fold -w 20| nl -n rz -s ‘ ‘| sed ‘/^/s/0/> fragment_/’| sed ‘s//\n/g’ > output_file_name”.

After the assembly of the trimmed Ion Torrent reads (T193-2) or trimmed Illumina reads (T195-1) using the “de novo assembly” tool with default parameters (mapping mode = created simple contig sequences), the generated k-mer sequences were mapped to the generated contig sequences of T193-2 and T195-1, respectively.

In parallel, the following trimmed reads were mapped to the contig sequences (default parameters but with an overlap of 90% and identity of 90%): (1) W52 Ion Torrent reads, (2) T193-2 Ion Torrent reads, (3) T195-1 Illumina reads. Moreover, all contig sequences with at least one mapped vector k-mer were subjected to BLASTN analyses versus (1) the sequence of pK2GW7_HSP_FT (MN379653), (2) *P. trichocarpa* contigs v3.0 (JGI Phytozome 2019; Tuskan et al. 2006) and (3) *P. tremula* contigs v1.1. (Lin et al. 2018; PopGenIE 2019).

### MinION data analysis

The primary data of the **first run of T193-2** was acquired with live base calling using the software MinKNOW (v2.2) for MinION from ONT, while sequencing in the MinION device with the aid of the MinIT. The MinKNOW produced FAST5 files which contain the raw signal data that was used as input for the Guppy basecaller (version v2.3.1.).

The primary data of the **second T193-2** run was acquired with live basecalling by the MinKNOW software from ONT (v2.2), while sequencing in the MinION device with the aid of an updated version of the MinIT (Release 19.01.1). Rebasecalling was not necessary.

The primary data of the **first T195-1 run** was acquired with live basecalling by the MinKNOW software from ONT, while sequencing in the MinION device with the aid of an updated version of the MinIT (Release 19.01.1). Rebasecalling was carried out using Guppy (v3.2.2).

The primary data of the **second T195-1 run** was initially acquired with live basecalling by the MinKNOW software from ONT, and the sequencing in the MinION device with the aid of the MinIT (Release 19.06.8). Rebasecalling was not necessary.

In order to improve the quality of the reads, the raw reads generated (FASTQ files) were further analysed with the Canu assembler (Koren et al. 2017) which is designed for the assembly of low coverage, long read data sets. The assembler first corrects the reads to improve the accuracy of the bases. Following this, a trimming step is performed to remove low quality reads before the assembly is performed. The output files (trimmed reads/contigs) were in FASTA format.

All BLASTN analyses with MinION trimmed reads were performed using the BLAST tools of CLC GWB v12.0.

## Results

### Sequencing of transgenic lines and mapping of short reads to the T-DNA vector sequence

The non-transgenic control line W52 and the transgenic lines T193-2 and T195-1 were sequenced using Ion Torrent PGM obtaining 6.8 × (W52), 14.9 × (T193-2) and 4.5 × (T195-1) haploid genome coverage, each (coverage of the trimmed reads; Suppl. file 2). Additional short reads (68 ×; 2 × 150 bp) were generated for T195-1 by Illumina HiSeq 4000 sequencing (Novogene, Beijing, China). Long reads of the two transgenic lines were generated by MinION nanopore sequencing (T193-2: run 1 with 3.32 ×, run 2 with 5.17 ×; 8.49 × haploid genome coverage in total; T195-1: run 1 with 11.74 ×, run 2 with 30.37 ×; 42.11 × in total). All short and long read data are available at the NCBI (SRA PRJNA576882; SRA PRJNA542603).

**Fig. 1:**
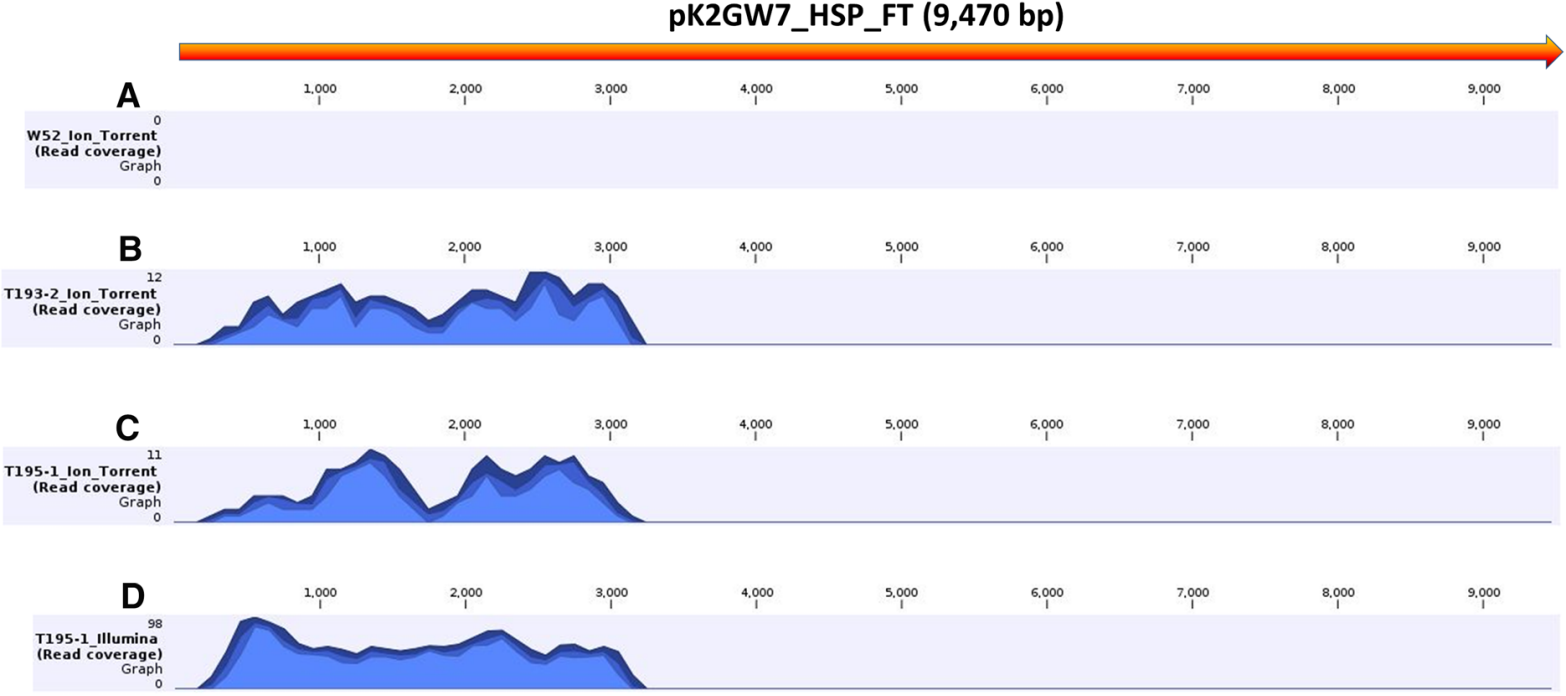
Coverage plots based on short read mappings to the T-DNA vector pK2GW7_HSP_FT (MN379653). **a** Mapping of Ion Torrent reads of W52 (wild type clone); **b** mapping of Ion Torrent reads of the transgenic line T193-2; **c** mapping of Ion Torrent reads of the transgenic line T195-1; **d** mapping of Illumina reads of the transgenic line T195-1

The trimmed Ion Torrent reads for all three genotypes and the trimmed Illumina reads for T195–1 were mapped to the nucleotide sequence of the T-DNA vector pK2GW7_HSP_FT (Genbank accession MN379653) using stringent parameters (Fig. 1). As expected, no reads of the wild type clone W52 mapped to the T-DNA vector (Fig. 1a). In the mappings of the transgenic lines (Fig. 1b–d), the 5-prime parts of the vector are covered by reads, thus suggesting the integration of a T-DNA insert in the respective lines. The T193-2 Ion Torrent reads cover the vector in the region from 245 to 3143 bp (Fig. 1b). The Illumina reads of T195-1 mapped contiguously to the vector in the region of 242 bp to 3145 bp (Fig. 1d), whereas the mapping of the T195-1 Ion Torrent reads was interrupted (251 bp to 1758 bp and from 1776 to 3141 bp; Fig. 1c) due to the low coverage of this data (4.5 ×). These results indicate that both transgenic lines nearly completely include the T-DNA insert (with partial left border and missing right border).

### Localizing the T-DNA insert in T193-2

Using the Ion Torrent data, the T-DNA insert of T193-2 was localised in the genome based on the identification of so called “chimeric reads”—reads in which one part mapped to the T-DNA vector and the other part showed a high similarity to the *P. tremula* and/or *P. trichocarpa* genome assembly (JGI Phytozome 2019; Lin et al. 2018; PopGenIE 2019; Sundell et al. 2015; Tuskan et al. 2006). In total, seven chimeric reads were identified for T193-2 (sequences in Suppl. file 3), 6 of them with at least 93% partial similarity to *P. trichocarpa* chromosome 13 and *P. tremula* contig Potra003542. One unexpected chimeric Ion Torrent read provided partial BLAST hits to *P. trichocarpa* chromosome 17 and to contig Potra003351 (sequence of read 68XWV:00229:02250 in Suppl. file 3). This chimeric read represents an individual chimera and is likely an artefact of the sequencing process as indicated by the low chimeric junction coverage. Moreover, the connection between the T-DNA vector and the *P. tremula* genome represented by this read could not be confirmed in any of the T193-2 trimmed MinION reads (Suppl. file 4A).

**Fig. 2:**
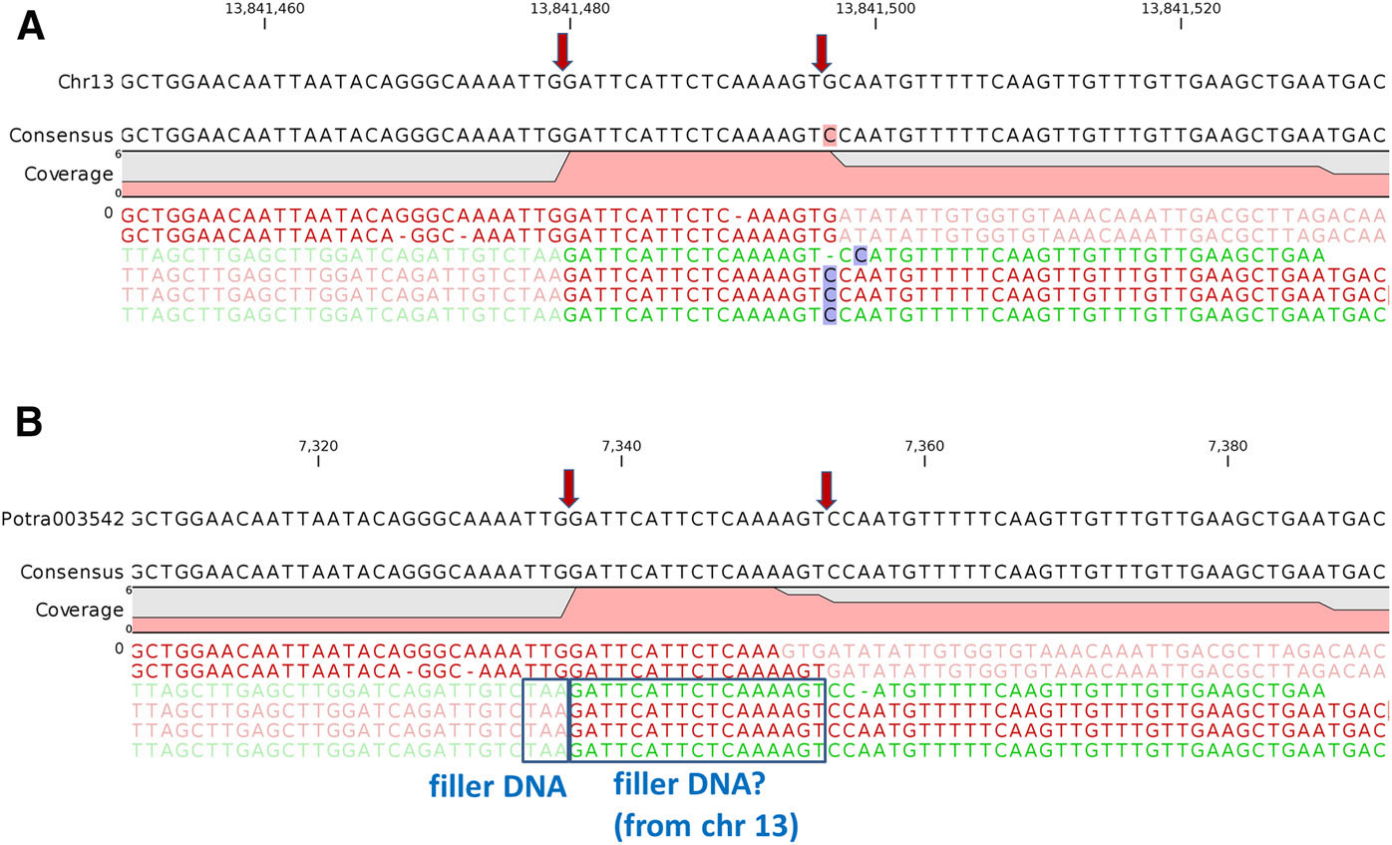
Potential integration sites of the T-DNA insert in T193-2 (red arrows). Mappings of chimeric reads to the nucleotide sequence of chromosome 13 of *P. trichocarpa* v3.0 (**a**) and of *P. tremula* v1.1 scaffold Potra003542 (**b**). Mapping was performed with CLC GWB (default parameters, but with 10% overlap and 90% similarity). Vector sequences flanking the integration sites in T193-2 are shown as transparent nucleotide sequences

Figure 2 shows the mappings of 6 chimeric reads to *P. trichocarpa* chromosome 13 (Fig. 2a) and *P. tremula* contig Potra003542 (Fig. 2b; cut-out enlargements). Based on this, two integration sites are possible at chromosome 13 (either between 13,841,479 and 13,841,480 or between 13,841,496 and 18,841,497; Fig. 2). The sequence between these sites is potentially integrated as a type of filler sequence in the transgenic insert. The two potential integration sites are located in the 3-prime UTR of Potri.013G125500 (annotated as Trigger Factor chaperone and peptidyl-prolyl cis/trans isomerase according PANTHER (JGI Phytozome 2019)).

To find out whether the T-DNA integration in T193-2 occurred in only one DNA strand (hemizygous) or in both, trimmed reads of T193-2 (Suppl. file 5A) and W52 (wild type control; Suppl. file 5B) were mapped to Potra003542 in parallel. The mapping results indicate that T-DNA integration was hemizygous in T193-2, since about half of the reads showed a continuous mapping to the genomic region, including the potential integration sites, and probably represent reads originating from the wild type strand. The other reads, on the other hand, were chimeric reads flanking the integration site and included vector parts (indicated by transparent sequence parts in the mappings; Suppl. file 5A).

**Fig. 3:**
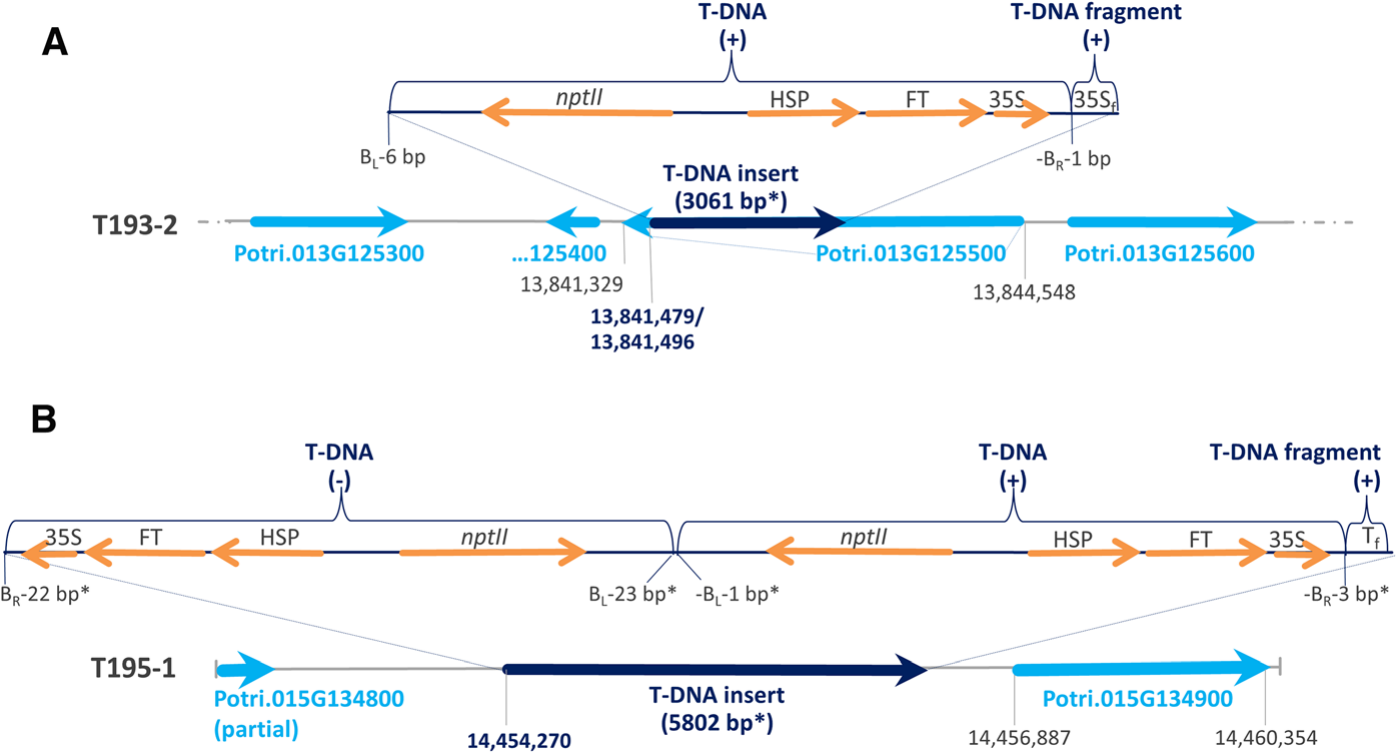
Localisation of T-DNA inserts in T193-2 (**a**) and T195-1 (**b**) based on long reads from MinION nanopore sequencing. The gene structures are derived from trimmed MinION reads (sequences in Suppl. files 6 and 7) that represent the top hits in the respective T-DNA vector BLAST (gene structures based on BLASTN analysis of the read sequences versus the *P. trichocarpa* genome assembly v3.0). Positions (in bp) in the gene maps are based on *P. trichocarpa* v3.0 (positions in bold represent T-DNA integration sites). *nptII*, *nptII* gene (NeoR/KanR; confers plant resistance to neomycin, kanamycin, and G418 (Geneticin^®^, plant selection marker); HSP, heat shock promoter; FT, *FLOWERING LOCUS T*; 35S, terminal 35S (CaMV 35S terminator); 35*S*_f_, fragment of 35S; *T*_f_, T-DNA fragment (provides a BLASTN hit to the T-DNA vector sequence in the T-DNA region; 433–805 bp); *B*_L_, left border; *B*_R_, right border; *, estimated bp values; (+), element at the plus strand in forward orientation; (−), element at the minus strand in reverse orientation. The sizes of the T-DNA inserts are based on NCBI alignment of the top read representing the vector-containing haplotype versus the read representing the wild type haplotype (sequences in Suppl. files 6 and 7)

An independent analysis of long reads from T193-2 generated by MinION nanopore sequencing in two runs (see above) was performed to validate the integration site identified by Ion Torrent and to analyse the structure of the T-DNA insert. The T-DNA vector sequence was analysed by BLASTN versus trimmed MinION reads and provided two hits with ≥ 90% identity (reads 1 and 2 in Suppl. file 6). These two reads represent the T-DNA-containing haplotype of T193-2. Figure 3a presents the gene structure in the genomic region of the T-DNA integration in T193-2 derived from the trimmed MinION read with highest BLAST score to the vector sequence (read 1 in Suppl. file 6). These results (Fig. 3a) confirm the Ion Torrent result that the T-DNA integration site is in the 3-prime UTR of Potri.013G125500 in T193-2. Interestingly, the T-DNA insert consists of a main T-DNA fragment and an additional partial fragment of terminal 35S (35S_f_; 3-prime part of terminal 35S; Fig. 3a).

BLASTN of the DNA sequence of *P. tremula* contig Potra003542 (including the integration sites, see above) identified one additional trimmed MinION read (read 3 in Suppl. file 6) without the T-DNA insert which represents the wild type DNA strand and supports the finding (see above) that the T-DNA integration is hemizygous.

### Localizing the T-DNA insert in T195-1

Based on the Ion Torrent data of T195-1, it was not possible to localise the T-DNA insert of T195-1 due to the too low coverage of the data (4.5-fold, see above). Only one chimeric read was identified which provided a partial BLAST hit to *P. trichocarpa* chromosome 2 and *P. tremula* scaffold Potra002148. This chimeric read is an individual chimera and is likely an artefact of the sequencing process as indicated by the low chimeric junction coverage (see also above). Moreover, the connection between the T-DNA vector and the *P. tremula* genome represented by this read could not be confirmed in any of the trimmed T195-1 MinION reads (Suppl. file 4B).

MinION nanopore sequencing allowed a localisation of the T-DNA insert. The T-DNA vector sequence was analysed by BLASTN versus trimmed MinION reads and provided several hits with ≥ 90% identity (hit with highest total BLAST score: read 1 in Suppl. file 7). Figure 3b presents the gene structure in the genomic region of the T-DNA integration in T195-1 derived from the sequence of read 1 (Suppl. file 7). The annotation of the non-vector parts of this read (based on BLASTN analyses versus the *P. trichocarpa* genome assembly v3.0 (JGI Phytozome 2019)) revealed that the T-DNA integration occurred at chromosome 15 in the intergenic region between Potri.015G134800 and Potri.015G134900. BLASTN analysis of the sequence of read 1 versus the T-DNA vector sequence revealed that the T-DNA insert (about 5802 bp in length) consists of three parts: (1) a reverse complement sequence of the T-DNA (identified by a BLASTN hit with 82% identity), (2) a forward sequence of the T-DNA (BLASTN hit with 95% identity) and (3) a partial sequence fragment of the T-DNA (*T*_*f*_ in Fig. 3b; BLASTN hit with 92% identity).

Unfortunately, the related *P. tremula* contig Potra000479 (showing the highest similarity to the broader genomic region flanking the integration site) shows an N-stretch in the integration site region. The BLASTN of the nucleotide sequence of Potra000479 versus the T195-1 trimmed MinION reads provided several additional reads that do not contain the T-DNA sequence. These reads represent the potential wild type haplotype. Altogether, these results confirm the hemizygous T-DNA integration.

### Systematic search for vector splinters in the transgenic lines

A global search for T-DNA or vector backbone splinters (Schouten et al. 2017) of at least 20 bp was performed in contig sequences of the transgenic lines which were assembled from short reads (Ion Torrent reads from T193-2; Illumina reads from T195-1). Considering a C-value of 440 Mbp for *P. tremula* (Siljak-Yakovlev et al. 2010) and an assembly size of 390 Mbp of version 1.1 of the *P. tremula* genome assembly at Popgenie (Lin et al. 2018; PopGenIE 2019), the assemblies are expected to be (nearly) complete (accumulated contig length of 443.9 Mbp in T195-1 and of 417.6 Mbp in T193-2).

**Fig. 4:**
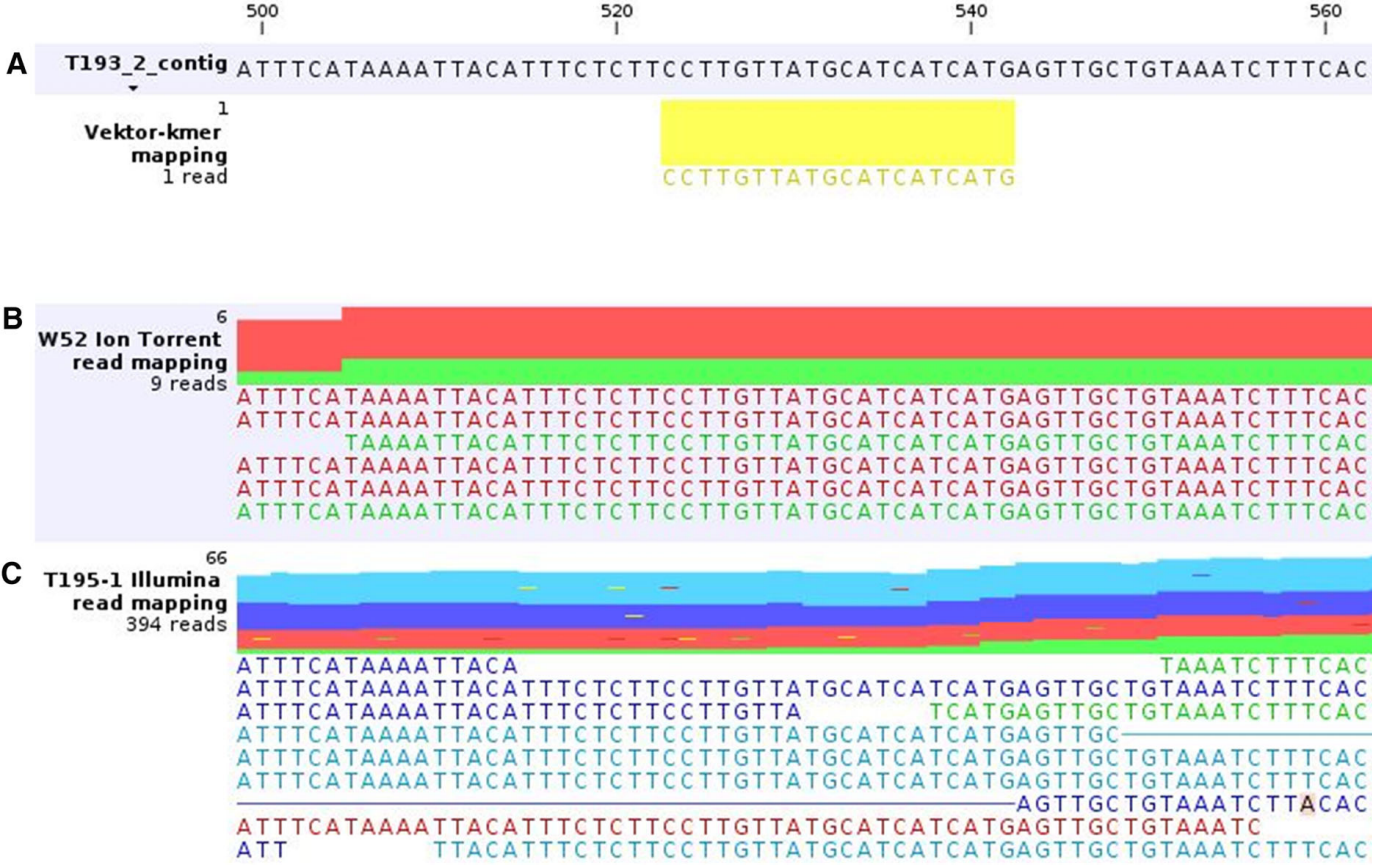
Mappings of vector k-mers (**a**), W52 wild type reads (**b**) and T195-1 reads (**c**) to T193-2 contig 201,495 (an enlargement of the track list is shown). The 20 bp region where one vector k-mer mapped to the contig sequence shows contiguous mappings of W52 reads as well as of reads from the other transgenic line, T195-1

To screen the contig sequences for T-DNA or vector backbone splinters/fragments, the complete DNA sequence of the T-DNA vector pK2GW7_HSP_FT (MN379653) was in silico digested to 20 bp k-mers (shift of 1 bp). The 9460 k-mers generated were mapped to the contig sequences of T193-2 or T195-1, respectively. Based on the mappings, 31 contigs from T193-2 and 23 from T195-1 with at least one k-mer hit each were selected and further analysed. Contigs that represent integrated T-DNA parts (contigs with contiguous mapping of vector k-mers) and contigs originating from genomic parts at the known integration borders (Suppl. file 8) were not further considered. For all remaining contigs, the related mapping track lists (see Materials and Methods) were inspected to check whether sequence stretches of mapped vector k-mers (Fig. 4a) are also present in the read mappings of the wild type genotype W52 (Fig. 4b) and of the other transgenic line (Fig. 4c). All these contigs (most of them with one internal k-mer hit) showed contiguous mappings of W52 reads and/or reads of the other transgenic line and/or contiguous BLASTN hits versus the *P. tremula* genome assembly (Lin et al. 2018; PopGenIE 2019) in the contig region with vector k-mer hits.

Thus, no T-DNA or vector backbone splinters/fragments could be identified outside the main integration sites of T193-2 or T195-1, respectively, when using the current database of short reads.

### Molecular analysis of second generation seedlings derived from crossings with *F*_1_-plants

**Fig. 5:**
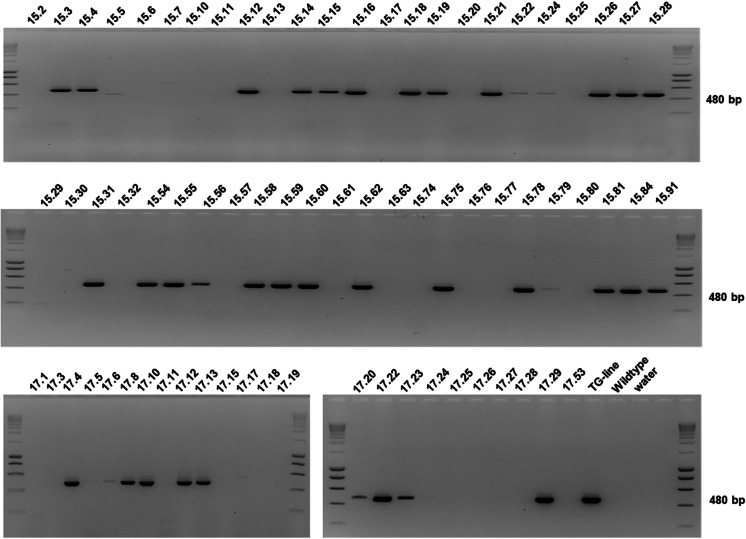
Molecular analysis of second generation-seedlings obtained from crossings between male HSP::*AtFT* T193-2 (*F*_1_ generation) and female wild type hybrid poplar (*P. tremula* x *P. tremuloides* Michx., clone Esch9) confirmed T-DNA presence in around 50% of the plants (second generation). PCR was carried out with *AtFT*-specific primers

Viable seeds (second generation) were obtained after crossings between a heat-induced early-flowering T193-2 seedling (♂, *F*_1_ generation) with a wild type hybrid poplar (*P. tremula x P. tremuloides* Michx., clone Esch9, ♀). In total, 72 seedlings germinated and were investigated for the presence of an *AtFT* gene fragment using PCR analyses. Results showed that of the 72 seedlings analysed, 38 tested positive for *AtFT*-PCR (indicating the presence of the early-flowering gene construct pHSP::*AtFT*), while 34 seedlings didn’t show any *AtFT*-PCR fragment (Fig. 5).

## Discussion

In this paper, we describe short and long read sequencing of two independent transgenic poplar lines (T195-1 and T193-2) carrying the heat-inducible *FLOWERING LOCUS T* gene from *A. thaliana* (pHSP::*AtFT*) and of the non-transgenic control clone W52 in order to unravel the genomic integration site(s) and the possible existence of T-DNA splinters and/or vector backbone sequences. In earlier investigations, both lines were genetically characterised using PCR and Southern blot analyses, and T-DNA was found to be integrated as single copy (Hoenicka et al. 2012, 2014, 2016).

Here, we confirmed the previous findings that just the T-DNA integrated in the genome of both transgenic lines and no vector backbone sequences could be detected (Hoenicka et al. 2014). For T193-2, we found the T-DNA integration in the gene locus of Potri.013G125500 (3-prime UTR) at chromosome 13 (Figs. 2 and 3a; Suppl. file 5), and for T195-1, in the intergenic region between Potri.015G134800 and Potri.015G134900 at chromosome 15 (Fig. 3b). As expected, both lines are hemizygous for the T-DNA insert. However, the T-DNA insertions consist of conglomerations of one (T193-2) or two T-DNA copies (T195-1), arranged as inverted repeat, together with a small T-DNA fragment without *AtFT* parts (Fig. 3). Inverted T-DNA repeats cannot be reliably detected by Southern Blot analyses. The integration of concatenated T-DNA fragments has also been reported in other studies (Gelvin 2003, 2017). Recently, two T-DNA insertions consisting of very complex T-DNA and vector backbone conglomerations have been identified in a transgenic *A. thaliana* line of the Salk collection (Jupe et al. 2019).

In the case of T193-2, the localisation of the T-DNA integration site was feasible using 14.9 × Ion Torrent data through the identification of chimeric reads. The 4.5 × coverage of the Ion Torrent data of T195-1 was too low for this purpose. Sequencing with MinION nanopore (T193-2: 8.5 ×; T195-1: 42.11 ×) enabled the resolution of the genomic structure of the T-DNA insert in both lines. Based on these results, we recommend haploid genome coverage of at least 10 × for localisation of the T-DNA insert of transgenic lines using high-throughput sequencing approaches.

The application of short chimeric NGS reads (Ion Torrent) to localise the T-DNA insert was hampered by the observed presence of individual chimera, which are likely artefacts of the sequencing process. The artefact nature of the individual chimeras is indicated by the low chimeric junction coverage in the Ion Torrent data and the lack of any chimeric junction coverage in the MinION data. Various types of artefacts including chimeras are commonly sequenced by NGS alongside the targeted RNA or DNA sequences. These artefacts are the result of experimental procedures, especially of library construction and PCR (Head et al. 2014; Lassmann et al. 2009; Schloss et al. 2011).

In this study, potential chromosomal translocations and exchanges that may be induced in T-DNA lines, as previously shown for *A. thaliana* (Jupe et al. 2019; Schouten et al. 2017), could not be analysed because a chromosome-level genome assembly of the wild type line W52 is thus far not available. Even the current version of the *P. tremula* reference genome assembly (individual Asp201) is not available at the chromosome level (Lin et al. 2018). Further studies using long-range sequencing technologies (nanopore sequencing, optical maps) are needed to compare the genome structures of the poplar transgenic lines with the related wild type line once a suitable reference sequence is available.

By applying conventional molecular methods (PCR, Southern blotting), many authors over the past 20 years have reported the transfer of additional T-DNA fragments and vector backbone sequences in addition to the transfer of the gene-of-interest in *Agrobacterium*-based transformation in different plant species (De Buck et al. 2000; Fladung 1999; Kononov et al. 1997; Kumar and Fladung 2002). All these events are often, but not necessarily, associated with unstable transgene expression (Kumar and Fladung 2001; Meza et al. 2002) and comprise a larger amount of unexpected partial T-DNA and/or vector integration. NGS is nowadays proposed and proven as an efficient method to also detect possible integrated T-DNA splinters and small vector backbone fragments in GM plants (Li et al. 2017; Park et al. 2015; Pauwels et al. 2015; Schouten et al. 2017).

We were not able to detect any T-DNA splinter or vector backbone sequences in the genome of the two male transgenic parent lines based on the current database of short NGS reads. Seedlings derived from crosses between the pHSP::*AtFT* transgenic male parents with a female wild type plant are therefore expected to be T-DNA splinter/vector backbone free. Thus, PCR analyses amplifying a partial T-DNA fragment are sufficient to determine whether the seedlings carry foreign DNA or not. Following the analysis of 72 *F*_2_-seedlings, we could clearly show that about half of the second generation-individuals still reveal the presence of the T–DNA, while the other half does not (Fig. 5), confirming previously published data obtained with the *F*_1_-generation (Hoenicka et al. 2014).

Some widely used and broadly accepted breeding methods induce large genetic changes that are often completely ignored. For instance, interspecific crossings between resistant and sensitive tree species have been carried out to obtain resistant hybrids (Brunet et al. 2013). However, this approach is very controversial, e.g. when non-native tree species are used that become invasive (Hoenicka and Fladung 2006). Furthermore, the offspring of interspecific crossings can show aberrant phenotypes. This effect, called incongruity (Hogenboom and Mather 1975), is the product of a miscommunication between genomes within an interspecific individual (Filler et al. 1994; Van Tuyl et al. 1991). Those seedlings showing aberrant phenotypes will normally be culled from the breeding programme and only those with good performance will be retained (Hoenicka et al. 2014).

But also mutational breeding has been found to induce stronger changes in plants than transgene insertion (Anderson et al. 2016; Batista et al. 2008). Other studies have revealed that mutagenesis can create more transcriptional changes in rice than transgenesis (Batista et al. 2008) and that the variation in transcriptomes, proteomes or metabolomes of many crops is lower in transgenic crops than in conventionally bred varieties (Baudo et al. 2006; Kogel et al. 2010; Lehesranta et al. 2005; Schnell et al. 2012).

However, with the currently ongoing “climate change”, i.e. observed environmental changes and increasing threats by biotic and abiotic stresses, forest tree species are confronted with serious problems that cannot be solved by conventional forest tree breeding. The long period of time necessary for forest tree species to reach the reproductive phase is a serious hindrance for their genetic improvement. Classical forest tree breeding has been unable to cope with many urgent challenges, e.g. the increasing spread of non-native illnesses threatening forests worldwide (Hoenicka and Fladung 2006). Dutch elm disease is a recent example for the urgency of implementation of new breeding strategies (Brunet et al. 2013). This illness is caused by the Asian fungi *Ophiostoma ulmi* and *O. novo*-*ulmi* and affects both European and American elm populations, and has not been resolved by classical breeding in over 100 years. Elm populations in Europe and North America have been decimated by this illness. Therefore, alternative breeding methods are highly needed to generate elm disease resistant trees. Faster breeding with e.g. GM early-flowering lines is a very promising approach in such cases.

However, the use of GM early-flowering lines for tree breeding requires the elimination of the early-flowering gene construct before release of these lines. Together with former studies (Hoenicka et al. 2014, 2016), this study once again confirms that the early-flowering trait can be applied to eliminate the foreign T-DNA. The generation of transgenic lines containing a single T-DNA copy is an important condition for transgene elimination in up to 75% of the offspring. Early-flowering systems open new possibilities for accelerating the breeding of forest tree species. Fast breeding and the selection of transgene-free plants, once the breeding process is accomplished, can represent an alternative breeding strategy, even under very restrictive biosafety regulations (Hoenicka et al. 2014).

In the EU, plant breeding with genetic transformation initiates a much more rigorous regulatory process than for mutagenesis (Schnell et al. 2012). However, the regulatory system in other countries, e.g. Canada and the USA, examines the novelty of traits in new crop varieties and not the processes used to generate them (Smyth and McHughen 2008). This approach should be seriously considered, especially if the obtained plants are transgene-free. The current biosafety regulation in the EU is not sustainable. Initially, genetically modified plants could be easily identified with PCR. However, the identification of transgene-free plants that have been improved with “new biotechnological methods” (e.g. genome editing) is not always possible. Although some methods have been proposed for the detection of gene-edited plants (reviewed in Grohmann et al. 2019; Schiemann et al. 2019), no methods are available for tracking the origin of transgene-free plants obtained from crossings with one early-flowering transgenic parent.

Data on putatively integration of T-DNA splinters or vector backbone sequences are needed by stakeholders of public risk assessors and regulators (Pauwels et al. 2015) to evaluate the regulatory status of putative transgene-free null segregants derived from self-fertilisation of a hemizygous transgenic plant or crosses with a non-GM plant. Holst-Jensen et al. (2016) highlighted the potential impact of NGS in risk assessment and traceability of GM plants. In two independent transgenic soybean lines, the insertion loci of the transgene and T-DNA-flanking regions identified by NGS could be confirmed by PCR and Sanger sequencing (Guo et al. 2016). In rice, Yang et al. (2013) detected additional unintended insertions compared to results from PCR and Southern blotting in two out of the three different independent transgenic lines investigated by NGS. In transgenic *A. thaliana*, Schouten et al. (2017) applied NGS to screen for genome-wide small mutations, possibly originating in the transformation process itself. However, only a few small mutations in the genomes of the five transgenic plants were identified and these were not correlated with the positions or number of T-DNA inserts. Instead, small and large deletions, as well as a translocation from another chromosome, were detected specifically at the T-DNA insert. Moreover, an additional tiny 50 bp T-DNA insert not previously detected by conventional PCR or Southern blotting was identified (Schouten et al. 2017).

In conclusion, we find that next and third generation sequencing technologies are highly sensitive approaches for the detection of T-DNA inserts. High coverage of NGS short reads is necessary to determine the T-DNA integration site based on chimeric reads. Long reads from third generation sequencing are very useful to unravel the genomic structure of T-DNA insertions. Further, NGS allows screening for potential T-DNA splinters or vector backbone sequences. The application of advanced long-range sequencing technologies will further promote the analysis of highly complex T-DNA insertions (concatemers, inverted repeats) as well as epigenetic modifications at the insertion site, and will support the identification of potential unanticipated genomic changes induced by T-DNA integration (Jupe et al. 2019).

## Electronic supplementary material

Below is the link to the electronic supplementary material.

Sequences of T-DNA vector primers (XLSX 9 kb)

Detailed information on Ion Torrent data of W52, T193-2, T195-1 (total number of trimmed reads, mean size, mean coverage and adapter information) (XLSX 11 kb)

Sequences of chimeric Ion Torrent reads (TXT 2 kb)

Results of BLASTN of one artefact chimeric Ion Torrent read (reference read) from T193-2 (**A**) and one from T195-1 (**B**) versus trimmed MinION reads (default BLAST parameters, but with decreased e-value of 0.001 and increased word size of 15). The composition of the chimeric Ion Torrent reads was analysed by BLASTN analyses of the read sequences versus *P. tremula* scaffolds (v1.1) at PopGenie (PopGenIE 2019) as well as versus the DNA sequence of the T-DNA vector pK2GW7_HSP_FT (MN379653). All MinION reads identified as BLAST hits map only to either the vector part or the *P. tremula* part of the Ion Torrent read; thus they do not confirm the connection between T-DNA vector and *P. tremula* genome as indicated by the chimeric Ion Torrent read (PPTX 836 kb)

Mappings of the trimmed Ion Torrent reads of the transgenic line T193-2 (**A**) and the wild type W52 (**B**) to the T-DNA integration sites at chromosome 13 (red arrows). The reads were mapped to the *P. tremula* scaffold Potra003542 (mapping using the CLC GWB with default parameters but 30% length fraction and 97% similarity fraction). Vector sequences flanking the integration sites in T193-2 are shown as transparent nucleotide sequences. Additional transparent nucleotide sequences originate from an Indel present in both the transgenic line T193-2 and the wild type W52 (TIFF 21941 kb)

Sequences of trimmed MinION reads from T193-2 (FASTA 104 kb)

Sequences of trimmed MinION reads from T195-1 (FASTA 55 kb)

Mappings of vector k-mers (**A**), W52 wild type reads (*B*) and T193-2 reads (**C**) to the nucleotide sequence of the T193-2 contig 52137 (an enlargement of the track list is shown). This contig is derived from one of the known border regions at the T–DNA integration site (TIFF 4072 kb)
