# Supplementary material for: Sequencing of two transgenic early-flowering poplar lines confirmed vector-free single-locus T-DNA integration
Source: Transgenic Res. 2020 Apr 30;29(3):321–37. doi: 10.1007/s11248-020-00203-0 (PMC7283205; doi:10.1007/s11248-020-00203-0)
Supplement: Supplementary file 4 — Results of BLASTN of one artefact chimeric Ion Torrent read (reference read) from T193-2 (A) and one from T195-1 (B) versus trimmed MinION reads (default BLAST parameters, but with decreased e-value of 0.001 and increased word size of 15). The composition of the chimeric Ion Torrent reads was analysed by BLASTN analyses of the read sequences versus P. tremula scaffolds (v1.1) at PopGenie (PopGenIE 2019) as well as versus the DNA sequence of the T-DNA vector pK2GW7_HSP_FT (MN379653). All MinION reads identified as BLAST hits map only to either the vector part or the P. tremula part of the Ion Torrent read; thus they do not confirm the connection between T-DNA vector and P. tremula genome as indicated by the chimeric Ion Torrent read (PPTX 836 kb) [file 11248_2020_203_MOESM4_ESM.pptx]

## Slide 1
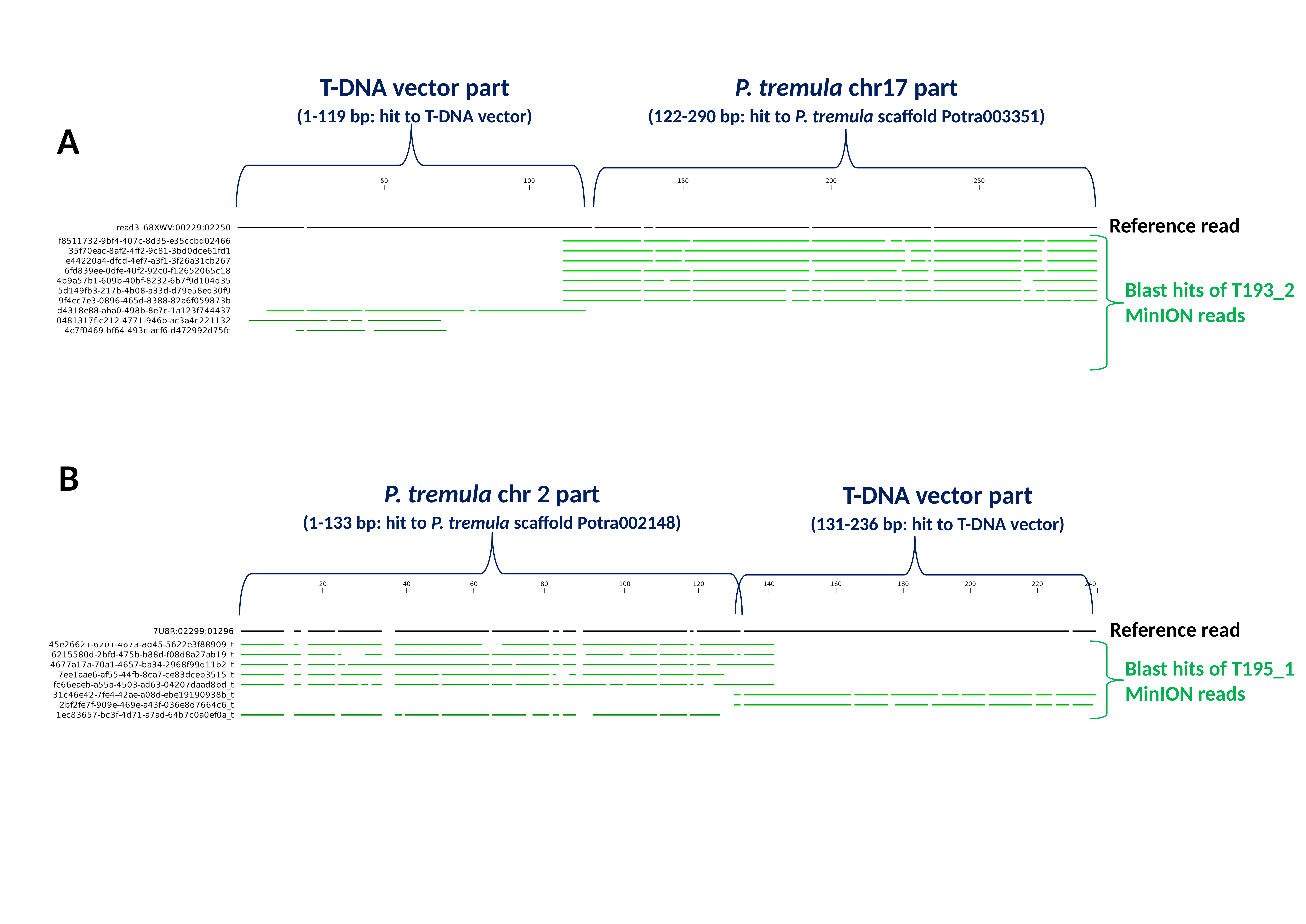

T-DNA vector part
(1-119 bp: hit to T-DNA vector)
P. tremula chr17 part
(122-290 bp: hit to P. tremula scaffold Potra003351)
A
Reference read
Blast hits of T193_2 MinION reads
B
P. tremula chr 2 part
(1-133 bp: hit to P. tremula scaffold Potra002148)
T-DNA vector part
(131-236 bp: hit to T-DNA vector)
Reference read
Blast hits of T195_1 MinION reads
