# Supplementary figures and images for: Sequencing of two transgenic early-flowering poplar lines confirmed vector-free single-locus T-DNA integration
Source: Transgenic Res. 2020 Apr 30;29(3):321–37. doi: 10.1007/s11248-020-00203-0 (PMC7283205; doi:10.1007/s11248-020-00203-0)

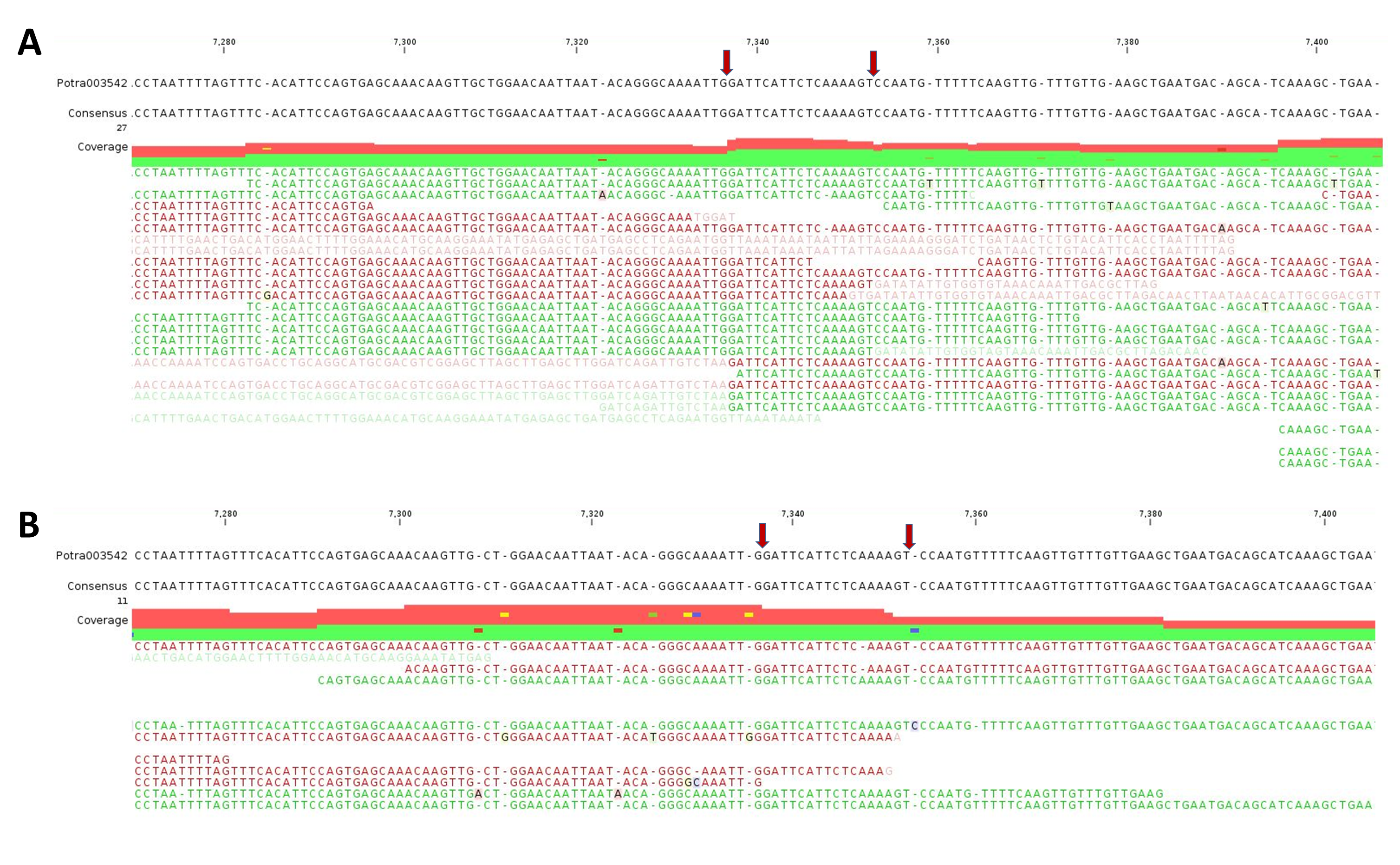

Supplement: Supplementary file 5 — Mappings of the trimmed Ion Torrent reads of the transgenic line T193-2 (A) and the wild type W52 (B) to the T-DNA integration sites at chromosome 13 (red arrows). The reads were mapped to the P. tremula scaffold Potra003542 (mapping using the CLC GWB with default parameters but 30% length fraction and 97% similarity fraction). Vector sequences flanking the integration sites in T193-2 are shown as transparent nucleotide sequences. Additional transparent nucleotide sequences originate from an Indel present in both the transgenic line T193-2 and the wild type W52 (TIFF 21941 kb) [file 11248_2020_203_MOESM5_ESM.tif]

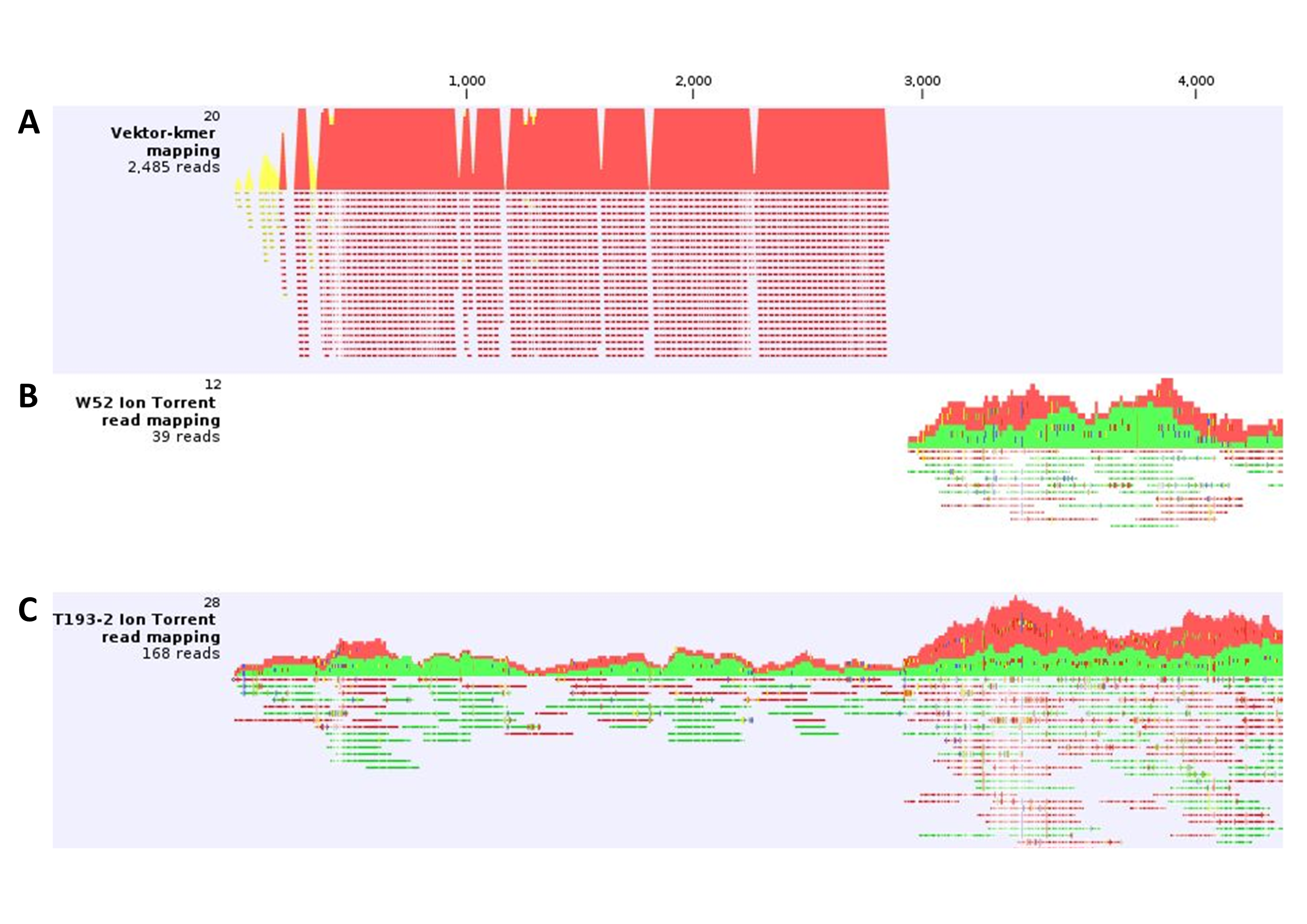

Supplement: Supplementary file 8 — Mappings of vector k-mers (A), W52 wild type reads (B) and T193-2 reads (C) to the nucleotide sequence of the T193-2 contig 52137 (an enlargement of the track list is shown). This contig is derived from one of the known border regions at the T–DNA integration site (TIFF 4072 kb) [file 11248_2020_203_MOESM8_ESM.tif]
